# A systematic review of palliative care knowledge, attitudes, and practices in Saudi Arabia: a global comparative analysis

**DOI:** 10.3389/fpubh.2026.1896172

**Published:** 2026-09-16

**Authors:** Sharifa M. Alasiry, Hatim Mohammed Alasiri, Abdulhakeem Ahmad Assiri

**Affiliations:** 1 Critical Care Nursing, College of Nursing, Majmaah University, Al Majma'ah, Saudi Arabia; 2 Health and Basic Sciences Research Center, Majmaah University, Al Majma'ah, Saudi Arabia; 3 Department of Palliative Care, King Fahad Medical City, Riyadh, Saudi Arabia

**Keywords:** attitudes, healthcare professionals, knowledge, palliative care, practices, Saudi Arabia

## Abstract

**Background:**

Palliative care is an important component of enhancing quality of life for patients with life-limiting illness. Despite increasing infrastructure capacity in Saudi Arabia, there is a significant gap in the clinical competence of healthcare professionals in palliative care. This systematic review attempted to assess the Knowledge, Attitudes, and Practices (KAP) of healthcare professionals in Saudi Arabia and compare them to international standards.

**Methods:**

A systematic review was conducted according to the guidelines of the PRISMA. Studies published between January 2012 and February 2026 were included, with a focus on the KAP of healthcare professionals regarding palliative care. A complete literature search was performed on the electronic databases (PubMed, Scopus, Web of Science, etc.). The review included cross-sectional and interventional studies of palliative care knowledge, attitudes, practices, and self-efficacy, quantitatively. The data from Saudi Arabia (9 studies) were compared to the international studies (21 studies) from other countries such as China, Taiwan, Ethiopia and Nigeria.

**Results:**

The research findings of studies carried out from 2012 to 2026 found low levels of knowledge on palliative care among health practitioners in Saudi Arabia. The problems included a lack of adequate knowledge, inadequate training, wrong beliefs about opioids, and poor end-of-life care plans. Global comparisons showed countries with an integrated and structured educational system, like Taiwan and China, had much higher competency levels; however, regions with *ad hoc* models of training, like Saudi Arabia, continued to show knowledge gaps. Major barriers were found to be lack of formal training in palliative care, opioid hesitancy and lack of implementation of end-of-life care protocols, especially in high acuity settings such as ICUs.

**Conclusion:**

The findings suggest that reliance on clinical exposure and *ad-hoc* training alone is insufficient to develop palliative care competency among healthcare professionals in Saudi Arabia. To improve workforce preparedness, structured and mandatory palliative care education should be integrated into undergraduate and postgraduate healthcare curricula. International evidence indicates that formal training programs are associated with improved knowledge, confidence, and palliative care practice. These findings suggest that educational and training deficiencies may contribute more substantially to competency gaps than cultural resistance alone.

## Introduction

The world landscape of healthcare is in the midst of a significant demographic and epidemiological transition. With the aging of our population and with the increase in prevalence of chronic illnesses as well as the rise in importance put on the quality of life, the need for palliative care has been apparent throughout the world (1). The World Health Organization defines palliative care as an approach to improve the quality of life for patients and families who are facing problems related to life-limiting illnesses by early identification, perfect assessment and treatment of physical, psychosocial, and spiritual pain (2). Even though this is a global mandate, integration into routine clinical practice is still a major challenge that highly depends on the preparedness, knowledge and attitudes of the frontline healthcare workers.

In the Kingdom of Saudi Arabia, this challenge is at the forefront of national health policy at the moment. Under the ambitious framework of Saudi Vision 2030, the country is implementing a massive healthcare transformation that has the goal of improving the quality of life for all citizens in the country and transforming the medical paradigm of healthcare to be holistic and patient-centered (3). Consequently, there has been a boom in physical infrastructure, such as the development of special cancer care facilities and palliative care units. However, there has been a critical paradox that has emerged: as the infrastructural capacity for the provision of palliative care expands, so does the critical gap of the human capital necessary to deliver it effectively, leaving a clear gap in workforce readiness (3).

Preliminary evidence over the last decade has indicated a lasting lack of clinical competency of Saudi healthcare professionals in end-of-life care. The origin of this clinical deficit begins at an early stage in the pipeline of education; for example, nursing students in Saudi Arabia have shown gross knowledge deficits; for example, 89.6% of this population wrongly believed that therapeutic administration of morphine invariably results in addiction (4). This foundation gap continues into professional practice, especially in high-acuity settings like Intensive Care Units (ICUs). ICU nurses and respiratory therapists in Saudi Arabia have much lower positive attitudes toward palliative care than physicians, often preferring curative and life-sustaining approaches, because of a lack of formal training and protocols (5).

Historically, these deficits in the Middle East have often been anecdotally attributed to cultural or religious barriers regarding death, dying and withdrawal of life-sustaining treatments. However, new data is coming out strongly contradicting this assumption. A recent study demonstrated that 52% of the Saudi public are supportive of their Islamic culture as a facilitator rather than a barrier to palliative care and highly value the concept of human dignity and symptom relief (6). Therefore, the major contributing factor is not cultural resistance but a systemic failure in the medical and nursing education that leaves the professionals with profound anxiety and a lack of self-efficacy when it comes to ethical decision-making and advanced pain management (3).

To properly identify the real reason for this “Confidence-Competence Gap” in Saudi Arabia, we need to look outside its borders. Examining national data alone runs the risk of misinterpreting large educational problems as smaller, local problems. By comparing the Saudi Arabian workforce metrics with different groups in the world, from East Asia with their well-organized education systems (7) to Africa with its under-resourced but improving education systems (8), we can find out those variables that are really the cause of competency in palliative care. Thus, this systematic review aims to compile more than 10 years of empirical data (between 2012 and 2026) to analyse Knowledge, Attitudes and Practices (KAP) concerning palliative care among healthcare professionals in Saudi Arabia, as this article traces the progress from early baseline assessments by Alamri in 2012 up until recent assessments conducted by Alzaidi et al. in 2026.

## Methods

### Study design and protocol

This study was designed as a systematic review of cross-sectional studies and interventional studies to evaluate the Knowledge, Attitudes and Practices (KAP) of healthcare professionals toward palliative care. The review was conducted and reported according to the Guidelines for reporting systematic reviews and meta-analyses of epidemiological studies (Preferred Reporting Items for Systematic Reviews and Meta-Analyses, or PRISMA) 2020. The main goal of this study was to consolidate data from Saudi Arabia and compare it with similar data from other countries to find structural and educational predictors of palliative care competency.

### Search strategy

A systematic and comprehensive literature search was conducted using the electronic databases PubMed/MEDLINE, Scopus, Web of Science, CINAHL, and Google Scholar. Studies published between January 2012 and February 2026 were searched to identify evidence regarding the knowledge, attitudes, practices, and self-efficacy of healthcare professionals toward palliative care. The final database search was performed on 28 February 2026. The search strategy combined Medical Subject Headings (MeSH) and free-text keywords related to palliative care, healthcare professionals, and competency outcomes. The main search string was: (“Palliative Care” OR “End-of-Life Care” OR “Hospice Care” OR “Terminal Care”) AND (“Knowledge” OR “Attitude” OR “Practice” OR “KAP” OR “Self-Efficacy”) AND (“Healthcare Professionals” OR “Nurses” OR “Physicians” OR “Intensive Care” OR “Students”). Geographical filters were applied to identify studies conducted in Saudi Arabia, the Middle East, Asia, and Africa. Additional relevant studies were identified through manual screening of the reference lists of eligible articles. The complete search strategies used for each electronic database are provided in Supplementary Table 2 to facilitate transparency and reproducibility of the literature search.

### Eligibility criteria

Studies were selected based on the following inclusion and exclusion criteria:

#### Inclusion criteria

***Population:*** Healthcare professionals (nurses, physicians, healthcare support workers) and medical or nursing students. In addition, one study assessing public perceptions of palliative care was included to provide contextual information regarding cultural attitudes and acceptance of palliative care. This study was analyzed separately and was not included in comparisons of professional knowledge, attitudes, or practices.

***Outcomes:*** Studies that quantitatively measured knowledge, attitude, practices and/or self-efficacy toward palliative care using validated questionnaires [e.g., Palliative Care Quiz for Nursing (PCQN), Frommelt Attitude Toward Care of the Dying (FATCOD) scale].

***Study design:*** Cross-sectional observational studies and pre/post-interventional studies.

***Language & timeframe:*** Published in English between January 2012 and February 2026.

#### Exclusion criteria

Studies that have focused solely on the clinical patient outcomes or prevalence of symptoms and not on provider competency. Purely qualitative research, case reports, editorials or previous systematic reviews/meta-analyses. Studies in which KAP data could not be distinctly extracted.

## PICOS framework

To ensure a transparent and structured approach to study selection, the eligibility criteria were developed according to the Population, Intervention/Exposure, Comparator, Outcomes, and Study Design (PICOS) framework. This framework guided the formulation of the review question, study selection, data extraction, and synthesis of evidence.

**Population (P):** The review included studies involving healthcare professionals directly or indirectly engaged in palliative care delivery, including physicians, nurses, respiratory therapists, healthcare support workers, and medical or nursing students. These populations were selected because they represent the principal workforce responsible for providing palliative care across various healthcare settings. In addition, one cross-sectional study evaluating public perceptions of palliative care in Saudi Arabia was included solely to provide contextual information regarding cultural attitudes and societal acceptance of palliative care. This study was analyzed separately and was not included in comparisons of healthcare professionals' knowledge, attitudes, or practices.

**Intervention/exposure (I):** The review considered studies evaluating palliative care knowledge, attitudes, practices, self-efficacy, educational exposure, formal palliative care training, or educational interventions designed to improve palliative care competency. Both observational assessments of existing competency and intervention studies evaluating educational programs or training initiatives were eligible for inclusion.

**Comparator (C):** Because most included studies were cross-sectional, a formal comparator was not mandatory for eligibility. Where applicable, comparisons included healthcare professionals with and without previous palliative care training, different professional groups (such as physicians vs. nurses), varying educational levels, different clinical practice settings (including intensive care units, oncology, primary care, and general hospital wards), and healthcare systems across different countries. These comparisons enabled the identification of factors associated with palliative care competency and facilitated international benchmarking.

**Outcomes (O):** The primary outcomes of interest were healthcare professionals' knowledge, attitudes, practices, and self-efficacy related to palliative care. These outcomes were measured using validated assessment instruments, including the Palliative Care Quiz for Nursing (PCQN), the Frommelt Attitude Toward Care of the Dying (FATCOD) scale, and other validated structured questionnaires. Secondary outcomes included barriers to palliative care implementation, predictors of competency (such as previous training and educational level), confidence in end-of-life care, opioid-related knowledge, communication skills, and ethical decision-making.

**Study design (S):** Eligible studies included quantitative cross-sectional observational studies and interventional studies evaluating educational or training interventions using pre–post designs. Only studies published in English between January 2012 and February 2026 were included. Qualitative studies, case reports, editorials, conference abstracts, narrative reviews, systematic reviews, meta-analyses, and studies that did not report quantitative outcomes related to knowledge, attitudes, practices, or self-efficacy were excluded.

## Study selection

The database search identified 350 records. After the removal of 15 duplicate records, 335 unique records remained for title and abstract screening. Following initial screening, 280 records were excluded because they did not meet the predefined inclusion criteria, including studies involving inappropriate populations, irrelevant outcomes, or unsuitable study designs. The full texts of these other 55 reports were attempted to be retrieved and checked for eligibility, 25 of which were excluded due to non-KAP focus, qualitative design and unvalidated assessment tools as the main reasons for exclusion. In the end, 30 studies that met all the criteria were included in this systematic review (Figure 1).

**Figure 1:**
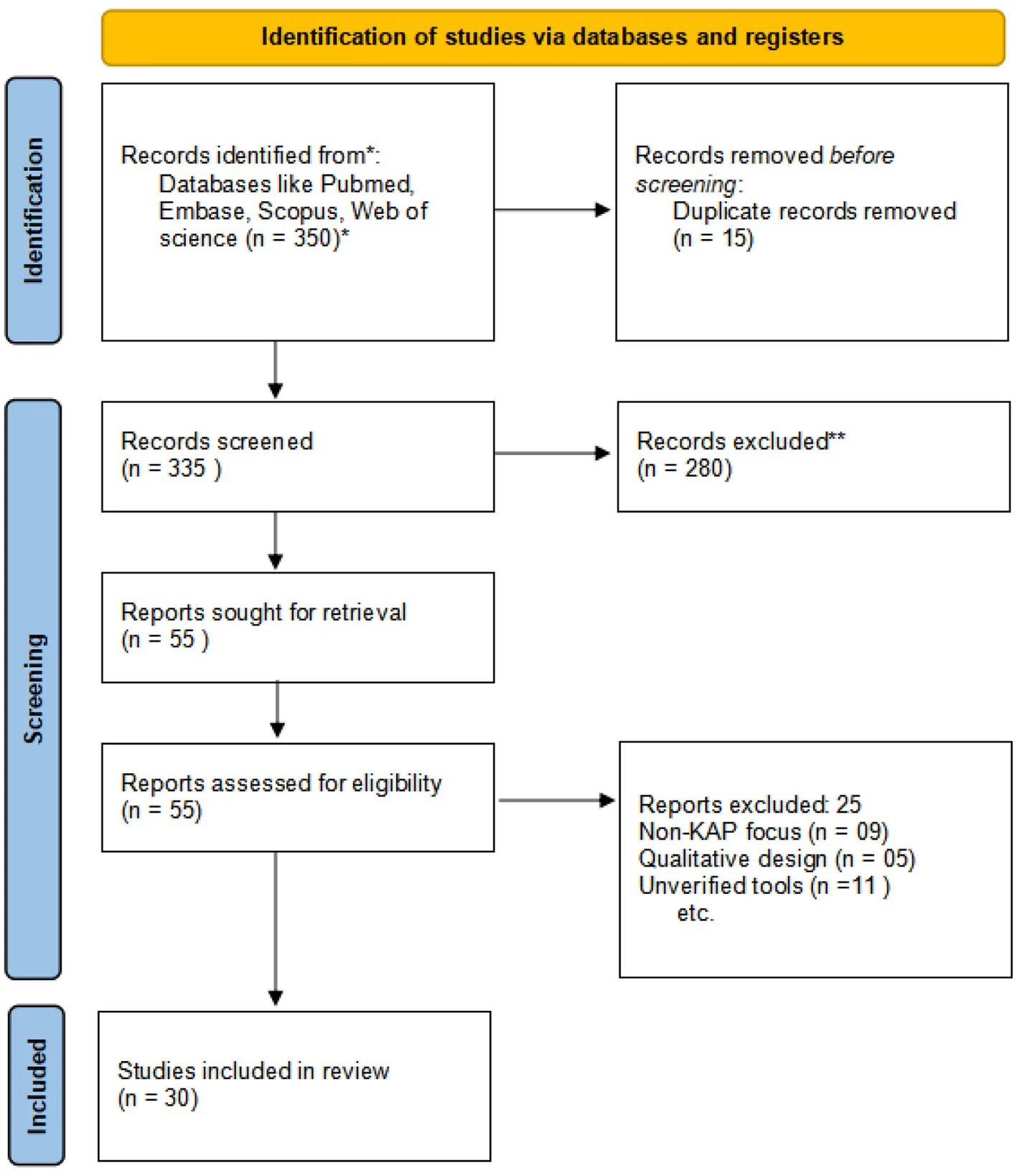
PRISMA flowchart.

## Data extraction

Data extraction was done systematically using a standardized charting form.

***Study characteristics:*** Author(s), year of publication, country/region, and study design.

***Participant demographics:*** Sample size (N), clinical setting (General Wards, ICU, Pediatrics, Oncology), and profession (Nurses, Physicians, Residents).

***Primary outcomes:*** Mean scores or percentages that indicated “Good/Optimal” Knowledge, “Positive/Favorable” Attitude, and “Proactive/Good Practice.

***Secondary outcomes:*** Major barriers identified, such as acute workload and opioid myths, plus major statistical predictors of competence like odds ratios for previous training or educational level.

## Risk of bias assessment

The methodological quality of the included studies was assessed using the Joanna Briggs Institute (JBI) Critical Appraisal Checklist for Analytical Cross-Sectional Studies, which is considered appropriate for evaluating cross-sectional research designs. The checklist evaluates several methodological domains, including sample selection, measurement validity and reliability, identification and management of potential confounding factors, and appropriateness of statistical analyses.

Two independent reviewers assessed the methodological quality of each included study. Any disagreements were resolved through discussion and consensus. Based on the overall appraisal, studies were categorized as high, moderate, or low methodological quality. Overall, most studies were found to be of moderate to high methodological quality, although some limitations related to convenience sampling, single-center designs, and self-reported outcome measures were identified.

## Data synthesis and benchmarking strategy

Because of substantial methodological heterogeneity among the included studies, a meta-analysis was not performed. Although several studies used commonly recognized instruments such as the Palliative Care Quiz for Nursing (PCQN) and the Frommelt Attitude Toward Care of the Dying (FATCOD) scale, important differences existed in participant populations, healthcare settings, outcome definitions, scoring systems, and reporting formats. In addition, several studies reported outcomes as categorical measures or provided insufficient statistical information for the calculation of comparable effect estimates. Therefore, quantitative synthesis was considered methodologically inappropriate, and a narrative synthesis approach was adopted.

In order to meet the study objectives, the results obtained were grouped into a Benchmarking Matrix. Saudi Arabian data (*n* = 9 studies) were summarized to form a national competency timeline. This schedule was then directly contrasted with a global group (*n* = 21 studies), stratified as a homogeneous set of profiles through the educational systems and geographic settings:

For comparative description, countries were classified based on similarities in the integration of education for palliative care, healthcare infrastructure, and competency outcomes. This classification was done merely to provide a basis for comparison in the discussion and analysis of the literature reviewed, and is not meant to suggest homogeneity in the healthcare systems of the different countries. There were marked differences in the resources, framework, and policies in the healthcare system.

Key predictors that included odds ratios, correlation coefficients, and regression estimates as documented across the research studies were used to investigate the relationship between training, knowledge, self-efficacy, attitudes, and palliative care practice.

## Results

### Study selection and characteristics

In this systematic review, 30 cross-sectional and interventional studies were used, the latest being published in 2026. The sample has a broad coverage of healthcare facilities, including general wards, intensive care units (ICUs), primary care, and pediatric units. The geographical areas covered in the cohort comprise Saudi Arabia (9 studies) and a large benchmark international cohort of 21 studies. The latter is of several regions, among which are East and Southeast Asia (China, Taiwan, South Korea, Malaysia), Central Asia (Mongolia), South Asia (India), the Middle East (Syria, Palestine, Iran), and Africa (Nigeria, Ethiopia, DR Congo).

### Knowledge, attitude, and practice of palliative care in Saudi Arabia (2012–2026)

Findings of research carried out in Saudi Arabia between 2012 and 2026 reveal consistent gaps in knowledge and skills regarding palliative care among medical practitioners in different healthcare environments (Table 1). The evidence presented covered a wide range of the national and regional settings, yet it remained mostly descriptive and indicated that research into palliative care in Saudi Arabia was primarily focused on recognizing the knowledge and training gaps rather than testing the effectiveness of interventions. In 2012, Alamri presented an alarming baseline according to which only 29.9% of medical residents were found to be knowledgeable of palliative care. Alzaidi et al. (3) similarly reported persistent knowledge gaps among healthcare professionals, with only 27.6% demonstrating adequate palliative care knowledge. The main problem appears to be related to shortcomings in undergraduate education. Khraisat (4) found that Saudi nursing students demonstrated poor knowledge levels, with a mean score of only 7.3 out of 20 on palliative care assessments. Furthermore, 89.6% of these students incorrectly believed that morphine use invariably results in addiction. These deficiencies appear to persist into postgraduate training, with Al-Drees et al. (9) reporting that 91.7% of medical residents had never received formal instruction in palliative care. High-acuity environments such as intensive care units (ICUs) are particularly affected by inadequate training. Qutob (5) reported that ICU nurses and respiratory therapists demonstrated less favorable attitudes toward palliative care compared with physicians. The lack of structured end-of-life care protocols and formal training contributed to a greater emphasis on curative and life-sustaining treatments rather than comfort-focused care.

**Table 1:** Characteristics and quality assessment of included studies from Saudi Arabia.

References	Region	Setting	Population (*N*)	Design	Assessment instrument	Key focus	Quality appraisal
Alzaidi et al. (3)	Saudi Arabia (National)	Various Hospitals	382 HCPs	Cross-Sectional persistent gaps	Structured KAP Questionnaire	General KAP and Self-Efficacy	Moderate
Qutob (5)	Saudi Arabia (Riyadh)	Adult ICU	203 Nurses/Physicians	Cross-Sectional	Structured Survey Instrument	ICU-specific barriers	Moderate
Jey (23)	Eastern Region	Primary Care	394 Nurses	Cross-Sectional	KAP Assessment Tool	Primary care readiness	Moderate
Alotaibi (24)	Saudi Arabia (National)	Hospitals	358 Support Workers	Cross-Sectional	Structured Questionnaire	Non-clinical staff KAP	Moderate
Alsolami (6)	Saudi Arabia (Jeddah)	Public/Community	488 General Public	Cross-Sectional	Public Perception Survey	Public perceptions and cultural attitudes toward palliative care†	Moderate
Al-Drees et al. (9)	Central Region	Tertiary Hospital	132 Residents	Cross-Sectional	Structured Questionnaire	Residency training gaps	Moderate
Khraisat (4)	Riyadh	University	165 Nursing Students	Cross-Sectional	PCQN	Undergraduate palliative care education	High
Abudari et al. (25)	Riyadh	Oncology Center	360 Nurses	Cross-Sectional	Structured Questionnaire	Multinational workforce perspectives	Moderate
Alamri (26)	Jeddah	University Hospital	87 Residents	Cross-Sectional	Structured Questionnaire	Baseline competency assessment	Moderate

### Global benchmarking: the “Universal Baseline”

The comparison between the findings in Saudi Arabia and international data will show that the deficits which are found are not an isolated situation but rather are a symptom of a “Universal Baseline” of healthcare systems with no mandated palliative curricula (Table 2). Knowledge ceiling is shared among most developing economies with an average of 45%. This is observed in Saudi Arabia (46%), Mongolia (44.6%), Ethiopia (40 to 47.9%), the non-cancer nursing wards in South Korea (48.6%), and in India (49.1) in the critical care nurses. Without formal and compulsory training, knowledge scores always lie in a range between 40 to 50.

**Table 2:** Characteristics and key findings of international benchmark studies.

References	Country	Setting	Population (*N*)	Assessment instrument	Main outcome measure	Key finding
He et al. (13)	China (Shanghai)	Adult ICU	224 ICU Staff	Structured KAP Questionnaire	Knowledge, Attitude, Practice	Formal training was associated with demonstrated palliative care practice.
Wei et al. (20)	China (Shanghai)	Tertiary Hospital	219 HCPs	Awareness and Attitude Survey	Awareness, Attitude	High support for palliative care despite limited formal training exposure.
Lu and Jin (14)	China (Multi-city)	Pediatric ICU	204 PICU Staff	Pediatric Palliative Care KAP Survey	Knowledge, Attitude, Practice	Reported among the highest knowledge levels identified in the review.
Swed et al. (11)	Syria	National	602 HCPs	Structured Questionnaire	Knowledge and Attitude	Significant knowledge deficits were observed in a conflict-affected healthcare environment.
Zhu (27)	China (Hunan)	Pediatrics	407 Pediatric HCPs	Structured Survey	Knowledge and Attitude	Moderate knowledge levels with generally favorable attitudes toward palliative care.
Azmi (10)	Malaysia	University Hospital	300 Physicians	Pain Management KAP Survey	Knowledge and Practice	Deficiencies in opioid-related knowledge were identified.
Kim et al. (19)	South Korea	General Wards	102 Nurses	Structured Questionnaire	Knowledge and Educational Needs	Moderate baseline palliative care knowledge among nurses caring for non-cancer patients.
Kim et al. (1)	Mongolia	Hospitals	168 Nurses	Self-Efficacy and Knowledge Survey	Knowledge, Self-Efficacy	Higher knowledge levels were associated with greater self-efficacy.
Walia et al. (22)	India	Critical Care	386 ICU Nurses	Structured Questionnaire	Knowledge, Practice, Barriers	Heavy workload and organizational factors were major barriers to palliative care delivery.
Zeru et al. (16)	Ethiopia (Tigray)	Hospitals	355 Nurses	Structured Questionnaire	Knowledge and Attitude	Formal training was associated with demonstrated knowledge levels.
Etafa et al. (18)	Ethiopia (Wollega)	Hospitals	342 Nurses	Structured Questionnaire	Knowledge and Attitude	Educational exposure was a significant predictor of knowledge.
Wu et al. (21)	China (Harbin)	Oncology	167 HCPs	Structured Survey	Knowledge, Attitude, Practice	Family-centered approaches to disclosure remained common in clinical practice.
Huang et al. (7)	Taiwan	Hospitals	412 HCPs	PCPCS-related Assessment	Knowledge, Attitude, Practice	Positive associations were identified among knowledge, attitudes, and clinical practice.
Farmani et al. (28)	Iran	Hospital	114 Nurses	KAP Questionnaire	Knowledge, Attitude, Practice	Moderate knowledge with generally favorable attitudes toward palliative care.
Ajisegiri et al. (8)	Nigeria	HIV Clinics	387 HCPs	Structured Questionnaire	Knowledge, Attitude, Practice	Formal training was associated with demonstrated clinical practice.
Ghoshal et al. (17)	India	Pediatrics	62 HCPs	Pre–Post Educational Assessment	Skills and Knowledge	Educational intervention demonstrated palliative care competencies.
Mukemo (2)	DR Congo	Hospitals	112 Nurses	Structured Questionnaire	Knowledge and Attitude	Limited training exposure was associated with poorer knowledge outcomes.
Gopal and Archana (29)	India	Tertiary Care	250 HCPs	Awareness Survey	Awareness and Attitude	Strong support for greater palliative care education within healthcare curricula.
Anteneh (30)	Ethiopia (Amhara)	Hospitals	359 Nurses	Structured Questionnaire	Knowledge and Attitude	Knowledge and attitudes varied according to educational background and experience.
Ayed (12)	Palestine	West Bank	96 Nurses	Structured Questionnaire	Knowledge and Attitude	Resource limitations contributed to deficiencies in palliative care preparedness.
Kassa et al. (31)	Ethiopia (Addis Ababa)	Hospitals	341 Nurses	Structured Questionnaire	Knowledge, Attitude, Practice	Positive attitudes were not consistently accompanied by adequate clinical practice.
Fadare et al. (15)	Nigeria	Tertiary Care	170 HCPs	Structured Questionnaire	Knowledge and Attitude	Knowledge deficits and substantial educational needs were identified.

The lack of pharmacological knowledge in opioids is a problem worldwide. Similar to Khraisat (4), who have determined the prevalence of addiction-myth beliefs in Saudi Arabia, Azmi (10) have reported that 66.3% of Malaysian physicians were not knowledgeable enough to safely prescribe opioids to cancer-related pain. Even worse off, areas where a high level of conflict or resource loss has been experienced, e.g., Syria (11) and Palestine (12), have experienced an even more pronounced knowledge collapse, with less than 20% of healthcare practitioners showing sufficient competency. On the same note, in Democratic Republic of Congo, Mukemo (2) noted that 90.2% of nurses were untrained, which resulted in a poor knowledge rate of 70.5% among them.

### The impact of structural education and interventions

The international dataset clearly shows that it is the structure of education—not just the simple exposure to clinical—that determines palliative care competence. Countries that have incorporated palliative care into their general medical practice tend to have better results (Table 3). Huang et al. (7) concluded that Taiwanese professionals had a moderate-to-high competency (63%), which was due to the establishment of Palliative Care Consultation Services (PCCS). Their study indicated that knowledge has a direct effect on both attitudes and practice using the structural equation modeling.

**Table 3:** Comparative findings on knowledge, attitudes, and practices (kap) in palliative care across regions.

Region	Knowledge status	Attitude status	Practice status	Major reported barriers
Saudi Arabia	Generally low to moderate knowledge levels (27%−46% demonstrating adequate knowledge)	Generally favorable attitudes toward palliative care, with reported confidence gaps in end-of-life communication and opioid use	Variable implementation of palliative care principles across settings; ICU studies reported greater emphasis on curative care	Limited formal undergraduate education, insufficient training opportunities, and concerns regarding opioid prescribing
China (PICU)	High knowledge levels reported	Moderately favorable attitudes	High levels of reported palliative care practice	Need for continued educational support and clinical integration
China/Taiwan (adult settings)	Moderate to high knowledge levels	Generally favorable attitudes and strong support for palliative care services	Practice positively associated with knowledge and training exposure	Communication challenges related to end-of-life discussions
Malaysia	Deficiencies identified in pain management and opioid-related knowledge	Mixed attitudes toward pain control and palliative care delivery	Variable clinical implementation	Limited training and inadequate pain assessment practices
Ethiopia/Nigeria	Low to moderate knowledge levels	Generally favorable attitudes toward palliative care	Practice demonstrated among healthcare professionals receiving formal training	Limited educational opportunities, workforce constraints, and resource limitations
Syria/Palestine	Substantial knowledge deficits reported	Mixed attitudes influenced by healthcare system challenges	Limited implementation of palliative care services	Healthcare system disruption, resource shortages, and conflict-related constraints

In comparison to international benchmark surveys (Table 3), Saudi Arabia had significantly low scores regarding knowledge about palliative care, with 27%−46% of respondents having sufficient knowledge in the reviewed studies. Nevertheless, such comparisons must be made with caution due to the differences between samples, healthcare settings, and tools used in these studies. At the same time, attitudes were generally positive but influenced by a fear of opioids and an inability to communicate truthfully. Practices in the Saudi Arabian healthcare were also inconsistent, especially in ICUs with an emphasis on curative treatment (7, 10–15).

While Saudi ICUs are struggling with the integration of palliative care, Chinese ICUs offer a model for success. Lu and Jin (14) found an outstanding score of 75% for trained Pediatric ICU personnel in 5 cities of China. Furthermore, He et al. (13) showed that the probability of proactive palliative practice increased 3.25-fold by formal training in adult ICUs in Shanghai.

Training has been seen to be effective around the world. In Ethiopia, Zeru et al. (16) reported that trained nurses were 3.48 times likely to have good knowledge. In Nigeria, Ajisegiri et al. (8) showed that previous training made the odds of good practice 5.2 times greater. An intervention in India, led by Ghoshal et al. (17) demonstrated immediate improvements in the pediatric care skills.

The findings from the predictors revealed that training had the highest and most stable relationship with palliative care competence (Table 4). Training demonstrated good practices by 5.2 times in Nigeria, proactive ICU practices by 3.25 times in China, and good knowledge by 3.5 times in Ethiopia. In addition, higher levels of education, more knowledge, and self-efficacy contributed to better attitudes and practice readiness, indicating that the weaknesses observed in Saudi Arabia could be addressed by integrating palliative care into the curricula, educating about opioids, and training on palliative care (2, 7, 8, 13, 16, 18, 19).

**Table 4:** Predictors of competence (global evidence).

Predictor	Evidence of impact	Source study
Training (general)	Increased odds of Good Practice by 5.2x.	Ajisegiri (Nigeria)
Training (ICU)	Increased odds of Proactive Practice by 3.25x.	He (China)
Training (nursing)	Increased odds of Good Knowledge by 3.5x.	Zeru (Ethiopia)
Education level	MSc/Degree nurses scored significantly higher than Diploma nurses.	Etafa (Ethiopia), Mukemo (DRC)
Knowledge on attitude	Higher knowledge directly increases positive attitude (β = 0.69).	Lu (China), Huang (Taiwan)
Knowledge on confidence	Higher knowledge significantly correlates with Self-Efficacy (*r* = 0.28).	Kim (Mongolia)

### Attitudes, cultural facilitators, and structural barriers

Across the world, healthcare workers demonstrate a “Confidence-Competence Gap” with many expressing high levels of support for palliative care even without appropriate training. In Shanghai, 95.4% of staff favored palliative care but only 21.5% had been trained (20). Similar trends of high positive attitudes and low knowledge were found in Nigeria (78.3% positive attitude) and Ethiopia (76% favorable attitude). Healthcare professionals are generally willing to provide end-of-life care and unfortunately are handicapped by a serious educational deficit.

In Mongolia, Kim et al. (1), they found that there is a direct correlation between low knowledge and low self-efficacy (*r* = 0.28, *p* < 0.001). This is responsible for the anxiety of Saudi professionals in taking ethical decisions and delivering bad news.

Cultural attitudes to death, it looks like, are not as much of a hurdle as they are often assumed to be. In Saudi Arabia, the public sees the Islamic culture as an enabler for palliative care as 52% of them think that the Islamic culture is in favor of palliative care practices as shown by Alsolami (6). In contrast, although cultural hesitancy to talk about death is mentioned in China (21), it is not viewed as an important hindrance.

Workload and institutional structural issues are important barriers to provision of palliative care. In India, Walia et al. (22) reported nurse's primary obstacles identified by critical care nurses to be “acute workload” and “attending to newly admitted patients.” These barriers are consistent with observations from Saudi ICUs where a lack of formal palliative care pathways means that critical care staff have to deal with acute, curative tasks at the expense of comfort care.

### Quality assessment results

The methodological quality of the 30 included studies was generally moderate to high. Six studies were classified as high quality, including those by Huang et al. (7), Lu and Jin (14), and He et al. (13), which achieved higher appraisal scores because of robust study designs, larger sample sizes, and the use of validated assessment instruments. Most studies (*n* = 17) were of moderate quality, including several studies from Saudi Arabia such as Alzaidi et al. (3) and Qutob (5), where limitations were primarily related to convenience sampling and limited adjustment for potential confounding factors. Seven studies were classified as low quality, mainly those conducted in conflict-affected or resource-constrained settings, where small sample sizes and incomplete methodological reporting increased the risk of bias (Supplementary Table 1).

## Discussion

### The illusion of clinical exposure: *ad-hoc* vs. structural education

The greatest finding in this systematic review is the failure of the “clinical exposure” model to develop competency in palliative care. In Saudi Arabia, healthcare professionals are highly dependent on *ad-hoc* learning on the job instead of the formal integration of palliative care into undergraduate education. Across studies published between 2012 and 2026, knowledge deficits remained a recurring finding among healthcare professionals in Saudi Arabia. However, direct temporal comparisons should be interpreted cautiously because studies differed in participant characteristics, clinical settings, and assessment instruments. Global benchmarking suggests that healthcare systems without structured and mandatory palliative care education frequently report low-to-moderate levels of palliative care knowledge. Similar findings have been reported in Ethiopia (40%−47.9%), Mongolia (44.6%), South Korea (48.6%), and India (49.1%). However, these comparisons should be interpreted cautiously because of differences in study populations, healthcare settings, and assessment instruments.

By contrast, the “East Asian Model” of education shows the effect of structural integration. In Taiwan, the development of Palliative Care Consultation Services (PCCS), the competency rate of palliative care reaches a moderate to high level (63%), and the structural equation modeling shows that a solid basic knowledge of palliative care is directly related to the improvement of attitudes and practices. The data shows that Saudi Arabia cannot depend on simply expanding the number of palliative care wards as it needs to make a critical overhaul of its undergraduate medical and nursing curricula, treating the knowledge deficits that originate in university settings. For example, Saudi nursing students have been evaluated and their mean scores have been shown to be as low as 7.3 out of 20 in knowledge test.

### Deconstructing the “ICU barrier”

The ICU is a site where the integration of palliative care into clinical practice is most resisted. In Saudi Arabia, ICU nurses and respiratory therapists have demonstrated less favorable attitudes toward palliative care and a greater preference for curative treatment approaches compared with physicians. This resistance is not limited to Saudi Arabia; it is a broader structural problem. In India, Walia et al. (22) identified that the biggest challenge in integrating palliative care within ICUs was the heavy burden of work and continuous flow of new admissions that compelled nurses to attend to acute care rather than end-of-life scenarios.

Nevertheless, international evidence suggests that this barrier can be overcome. In China, Lu and Jin (14) showed that PICU personnel could achieve impressive knowledge scores (75%) when given specialized training in palliative care. Further, He et al. (13) reported that training adult ICU staff in Shanghai increased the odds for proactive palliative care by 3.25 times. These findings suggest that limited specialized training and insufficient exposure to palliative care principles may contribute to lower readiness for palliative care delivery in ICU settings.

### The global “concerns regarding opioid prescribing”

Pain control is the foundation of palliative care; however, opioid hesitancy is a major barrier worldwide. In Saudi Arabia, Khraisat (4) reported an alarming lack of knowledge in opioid prescribing when 89.6% of nursing students thought that therapeutic morphine would always lead to addiction. This pharmacological ignorance is not limited to Saudi Arabia, extending across borders. In Malaysia, Azmi (10), who interviewed 66.3% of physicians, found that they are not well-versed in safe prescription practices for opioids. Even in India, despite targeted interventions, Ghoshal et al. (17) found that professionals are still too conservative and use morphine when all other analgesics have failed. This is widespread concerns regarding opioid prescribing that further emphasizes the need for targeted education as well as comprehensive national prescribing guidelines that protect and empower healthcare practitioners from regulatory repercussions in their treatment of severe pain.

### Cultural receptivity vs. professional insecurity

Conservative Islam has often been put in opposition to palliative care when it comes to truth telling and withdrawal of life support, but this data does not support this assumption. Alsolami (6) found that it is believed that 52% of the Saudi public believe that their culture and religion supports palliative care as it relates to dignity and relief from symptoms. The findings suggest that professional knowledge gaps, limited training opportunities, and low self-efficacy may contribute substantially to palliative care competency challenges in Saudi Arabia, alongside broader cultural and healthcare-system factors. Several studies reported lower confidence and self-efficacy among healthcare professionals when addressing ethical decision-making, end-of-life communication, and symptom management.; these findings are similar to those in Mongolia where Kim et al. (1) found a direct correlation (*r* = 0.28) between low knowledge and low clinical confidence. Moreover, “family-centric” truth-telling in which the diagnosis is concealed from the patient is also present in China (21). This suggests that such practices are more a matter of concerns of collective societies grappling with complicated medical ethics than an inherent obstacle posed by religion.

### The transformative power of targeted interventions

International data demonstrate a clear path to improvement, evidenced in spite of universal deficits in knowledge and skills in palliative care. The Training Multiplier effect has been reported everywhere regardless of resource levels. In Ethiopia, targeted training increased the odds for nurses to have good knowledge of palliative care by 3.48 times; while in Nigeria, training increased the odds of good clinical practice by 5.2 times. In China, where there was very little formal training (only 21.5% of staff) basic support for palliative care was almost universal at 95.4%—which shows that healthcare professionals are willing and eager recipients of educational change.

Compared with several international benchmark studies, Saudi Arabia continues to demonstrate important gaps in palliative care knowledge, training, and practice. Given the ongoing healthcare transformation under Saudi Vision 2030 and the expansion of palliative care services, strengthening structured educational programs and integrating palliative care into undergraduate and postgraduate curricula may help improve workforce preparedness and competency.

## Conclusion

The current transformation of health care services within the context of Saudi Vision 2030 calls for the development of palliative care services that must be complemented by adequate readiness of the workforce. Through this systematic review, gaps in terms of knowledge, training, and practice relating to palliative care have been found among health care providers in Saudi Arabia despite having positive attitudes toward the concept of palliative care.

International evidence suggests that structured educational programs, competency-based training, and clear clinical guidelines are associated with demonstrated palliative care knowledge, confidence, and practice. These findings support the integration of standardized palliative care curricula into undergraduate and postgraduate healthcare education programs in Saudi Arabia. Strengthening professional training may contribute to demonstrated delivery of patient-centered and end-of-life care in alignment with national healthcare priorities.

## Data Availability

The original contributions presented in the study are included in the article/Supplementary material, further inquiries can be directed to the corresponding author.
