# Supplementary material for: A systematic review of palliative care knowledge, attitudes, and practices in Saudi Arabia: a global comparative analysis
Source: Front Public Health. 2026 Sep 16;14:1896172. doi: 10.3389/fpubh.2026.1896172 (PMC13624088; doi:10.3389/fpubh.2026.1896172)
Supplement: Supplementary file 1 [file Supplementary_file_1.pdf]

Seven studies were classified as low quality, mostly those performed in conflict-affected or resource-constrained settings, in which small sample sizes and incomplete reporting of methods led to an increased risk of bias (**Supplementary Table 1**)."

Supplementary Table 1

Detailed JBI Critical Appraisal Assessment of Included Studies

**Study**	**Q1 Inclusion Criteria**	**Q2 Study Subjects & Setting**	**Q3 Exposure Measurement**	**Q4 Objective Criteria**	**Q5 Confounders Identified**	**Q6 Confounder Management**	**Q7 Outcome Measurement**	**Q8 Statistical Analysis**	**Overall Quality**
Alamri (2012)	Y	Y	Y	Y	N	N	Y	Y	Moderate
Abudari et al. (2014)	Y	Y	Y	Y	N	N	Y	Y	Moderate
Kassa et al. (2014)	Y	Y	Y	Y	Y	N	Y	Y	Moderate
Fadare et al. (2014)	Y	Y	Y	Y	N	N	Y	Y	Moderate
Ayed et al. (2015)	Y	Y	Y	Y	N	N	Y	Y	Moderate
Gopal et al. (2016)	Y	Y	Y	Y	N	N	Y	Y	Moderate
Anteneh et al. (2016)	Y	Y	Y	Y	Y	N	Y	Y	Moderate
Khraisat et al. (2017)	Y	Y	Y	Y	Y	Y	Y	Y	High

Mukemo et al. (2017)	Y	Y	U	Y	N	N	Y	Y	Low
Ghoshal et al. (2018)	Y	Y	Y	Y	Y	Y	Y	Y	High
Farmani et al. (2019)	Y	Y	Y	Y	N	N	Y	Y	Moderate
Ajisegiri et al. (2019)	Y	Y	Y	Y	Y	Y	Y	Y	High
Huang et al. (2019)	Y	Y	Y	Y	Y	Y	Y	Y	High
Al-Drees et al. (2019)	Y	Y	Y	Y	N	N	Y	Y	Moderate
Zeru et al. (2020)	Y	Y	Y	Y	Y	Y	Y	Y	Moderate
Etafa et al. (2020)	Y	Y	Y	Y	Y	N	Y	Y	Moderate
Kim et al. (2020)	Y	Y	Y	Y	N	N	Y	Y	Moderate
Wu et al. (2020)	Y	Y	Y	Y	N	N	Y	Y	Moderate
Walia et al. (2020)	Y	Y	Y	Y	N	N	Y	Y	Moderate

Azmi et al. (2022)	Y	Y	Y	Y	N	N	Y	Y	Moderate
Alsolami et al. (2023)	Y	Y	Y	Y	N	N	Y	Y	Moderate
Zhu et al. (2023)	Y	Y	Y	Y	N	N	Y	Y	Moderate
Lu & Jin (2024)	Y	Y	Y	Y	Y	Y	Y	Y	High
Swed et al. (2024)	Y	Y	U	Y	N	N	Y	Y	Low
Alotaibi (2024)	Y	Y	Y	Y	N	N	Y	Y	Moderate
Jey (2024)	Y	Y	Y	Y	N	N	Y	Y	Moderate
Wei et al. (2025)	Y	Y	Y	Y	N	N	Y	Y	Moderate
He et al. (2025)	Y	Y	Y	Y	Y	Y	Y	Y	High
Qutob et al. (2025)	Y	Y	Y	Y	N	N	Y	Y	Moderate
Alzaidi et al. (2026)	Y	Y	Y	Y	N	N	Y	Y	Moderate

Abbreviations: Y = Yes; N = No; U = Unclear.

Supplementary Table S2. Database-specific search strategies used for the literature search

**Database**	**Search Strategy**	**Filters Applied**
**PubMed/MEDLINE**	("Palliative Care"[Mesh] OR "Palliative Care" OR "End-of-Life Care" OR "Hospice Care" OR "Terminal Care") AND ("Knowledge" OR "Attitude" OR "Practice" OR "KAP" OR "Self-Efficacy") AND ("Healthcare Personnel"[Mesh] OR "Healthcare Professionals" OR Nurses OR Physicians OR "Respiratory Therapists" OR "Medical Students" OR "Nursing Students")	English language; January 2012–February 2026; Human studies
**Scopus**	TITLE-ABS-KEY ("palliative care" OR "end-of-life care" OR "hospice care" OR "terminal care") AND TITLE-ABS-KEY (knowledge OR attitude OR practice OR KAP OR self-efficacy) AND TITLE-ABS-KEY ("healthcare professionals" OR nurses OR physicians OR "respiratory therapists" OR "medical students" OR "nursing students")	English language; January 2012–February 2026
**Web of Science**	TS=("palliative care" OR "end-of-life care" OR "hospice care" OR "terminal care") AND TS=(knowledge OR attitude OR practice OR KAP OR self-efficacy) AND TS=("healthcare professionals" OR nurses OR physicians OR "respiratory therapists" OR "medical students" OR "nursing students")	English language; January 2012–February 2026

**CINAHL**	(MH "Palliative Care+") OR ("Palliative Care" OR "End-of-Life Care" OR "Hospice Care") AND (Knowledge OR Attitude OR Practice OR KAP OR Self-Efficacy) AND (MH "Health Personnel+" OR Nurses OR Physicians OR "Healthcare Professionals" OR "Medical Students" OR "Nursing Students")	English language; January 2012–February 2026
**Google Scholar**	("Palliative Care" AND Knowledge AND Attitude AND Practice AND "Healthcare Professionals") OR ("Palliative Care" AND Nurses) OR ("End-of-Life Care" AND Physicians)	English language; January 2012–February 2026; first 200 most relevant records screened
